# Operationalization of diagnostic criteria of DSM-5 somatic symptom disorders

**DOI:** 10.1186/s12888-017-1526-5

**Published:** 2017-11-07

**Authors:** Nana Xiong, Yaoyin Zhang, Jing Wei, Rainer Leonhart, Kurt Fritzsche, Ricarda Mewes, Xia Hong, Jinya Cao, Tao Li, Jing Jiang, Xudong Zhao, Lan Zhang, Rainer Schaefert

**Affiliations:** 1 Department of Psychological Medicine, Peking Union Medical College Hospital, Chinese Academy of Medical Sciences & Peking Union Medical College, Beijing, 100730 China; 2 Department of Psychosomatic, Sichuan Provincial People’s Hospital, Chengdu, Sichuan People’s Republic of China; 3 Institute of Psychology, University of Freiburg, 79085 Freiburg, Germany; 4 Department of Psychosomatic Medicine and Psychotherapy, University Medical Centre Freiburg, Freiburg, Germany; 5 Department of Psychology, Philipps University of Marburg, Gutenbergstr. 18, 35032 Marburg, Germany; 6 Department of Psychosomatic Medicine, Dongfang Hospital, School of Medicine, Tongji University, Shanghai, 200120 China; 7 Mental Health Centre, West China Hospital, Sichuan University, Chengdu, 610041 Sichuan People’s Republic of China; 8 Department of Psychosomatics, Medical Division, University Hospital Basel, Basel, Switzerland

**Keywords:** DSM-5, Somatic symptom disorder, Somatoform disorders, Multiple somatic symptoms, China

## Abstract

**Background:**

The aim of this study was to test the operationalization of DSM-5 somatic symptom disorder (SSD) psychological criteria among Chinese general hospital outpatients.

**Methods:**

This multicenter, cross-sectional study enrolled 491 patients from 10 general hospital outpatient departments. The structured clinical “interview about cognitive, affective, and behavioral features associated with somatic complaints” was used to operationalize the SSD criteria B. For comparison, DSM-IV somatoform disorders were assessed with the Mini International Neuropsychiatric Interview plus. Cohen’s к scores were given to illustrate the agreement of the diagnoses.

**Results:**

A three-structure model of the interview, within which items were classified as respectively assessing the cognitive (B1), affective (B2), and behavioral (B3) features, was examined. According to percentages of screening-positive persons and the receiver operator characteristic (ROC) analysis, a cut-off point of 2 was recommended for each subscale of the interview. With the operationalization, the frequency of DSM-5 SSD was estimated as 36.5% in our sample, and that of DSM-IV somatoform disorders was 8.2%. The agreement between them was small (Cohen’s к = 0.152). Comparisons of sociodemographic features of SSD patients with different severity levels (mild, moderate, severe) showed that mild SSD patients were better-off in terms of financial and employment status, and that the severity subtypes were congruent with the level of depression, anxiety, quality of life impairment, and the frequency of doctor visits.

**Conclusions:**

The operationalization of the diagnosis and severity specifications of SSD was valid, but the diagnostic agreement between DSM-5 SSD and DSM-IV somatoform disorders was small. The interpretation the SSD criteria should be made cautiously, so that the diagnosis would not became over-inclusive.

**Electronic supplementary material:**

The online version of this article (10.1186/s12888-017-1526-5) contains supplementary material, which is available to authorized users.

## Background

The diagnostic category of somatoform disorders (SD) in the *Diagnostic and Statistical Manual of Mental Disorders, Fourth Edition* (DSM-IV) [1] has been revised and replaced with somatic symptom disorder (SSD) in DSM-5 [2]. Besides the requirement of persistent one or more distressing somatic symptoms, the diagnostic focus has shifted from whether symptoms were medically unexplained to positive psycho-behavioral criteria, including disproportionate thoughts, feelings and behaviors related to somatic symptoms or health concerns [2]. According to the DSM-5, “the prevalence of SSD in the general adult population may be approximately 5%-7%.” Concerns were raised that, if handled improperly, a vast group of people might be mislabeled with mental disorders [3]. In addition, for decades, Chinese people have been believed to be more likely to express somatic symptoms than their Western counterparts [4, 5]. Past studies have confirmed that distressing somatic symptoms were common in Chinese general hospital outpatients [6, 7]. However, it is unknown to what extent the new SSD concept, which focuses more on psycho-behavioral characteristics, can be applied to Chinese hospital outpatients.

Nevertheless, instruments to establish the diagnosis of SSD were still lacking, especially regarding the assessment of the psycho-behavioral symptoms. Up to now, the Chinese version of the Structured Clinical Interview for DSM-Disorders, fifth edition (SCID-5) still needs to be validated. Among the known measurements, the Whiteley Index (WI-14 or WI-7) was previously employed to operationalize SSD [8, 9]. However, the WI measures health anxiety, reflecting only the affective features specified in the SSD criteria. Two new self-reported questionnaires were also developed and validated to assess the psycho-behavioral criteria, including the SSD-12 [10, 11] and the Somatic Symptoms Experiences Questionnaire [12]. However, in terms of establishing a diagnosis, self-rated questionnaires are believed to be less reliable than clinical interviews, in which subjects have the opportunity to ask the meanings of unfamiliar words [13].

Therefore, this is the first study using a structured clinical interview, the “interview about cognitive, affective, and behavioral features associated with somatic complaints” (ICAB), to investigate different operationalization of the three dimensions of the SSD criteria B, “disproportionate and persistent thoughts about”, “persistently high level of anxiety about”, and “excessive time and energy devoted to” their symptoms [2]. Since these items are general and ambiguous and might only represent a part of the numerous psycho-behavioral features of somatizing patients reviewed in the past, the interview intended not only to capture and operationalize the three dimensions specified in DSM-5 (such as by assessing rumination and catastrophizing thoughts, illness worries, frequent bodily self-observation, and health care utilization), but also to broaden the diagnostic basis by including some more specific psycho-behavioral characteristics of somatizing patients (such as somatic illness beliefs, feeling of injustice, desperation because of symptoms, and negative self-concept of bodily weakness) [8, 14]. In a current cohort study, the ICAB has shown relevance and predictive value for somatoform symptoms [15].

In addition, the diagnostic agreement between the DSM-5 SSD and the DSM-IV SD was small in previous studies [8, 9]. When the clinical interview ICAB was adopted to assess SSD in Chinese, its agreement with SD remained unclear. Furthermore, with limited instruments, few studies have been conducted to operationalize the SSD severity levels [9]. Thus, using a combination of assessment instruments, we aimed to test the following research questions among a sample of Chinese general hospital outpatients: 1) to operationalize the diagnostic criteria and severity specifications of DSM-5 SSD; and 2) to compare the frequencies and agreement of DSM-5 SSD and DSM-IV somatoform disorder.

## Methods

### Study design and setting

This is a secondary analysis of data collected within a multicenter cross-sectional study between February 1, 2011, and October 30, 2012 [16], which was conducted in 10 outpatient clinics of tertiary hospitals in Beijing, Shanghai, Chengdu, and Kunming (located at the north, southeast, and southwest of China). Among them, the neurology and gastroenterology departments were chosen to represent the modern biomedical settings, the Traditional Chinese Medicine (TCM) departments were selected to represent the traditional medical settings, and the psychological medicine departments were chosen to represent the psychosomatic medical settings. Patients from the above three medical settings were supposed to be evenly recruited. On randomly assigned days, outpatients were consecutively informed about the study and invited to participate.

All participants were assessed by the somatic symptom scale of the Patient Health Questionnaire (PHQ-15), thereby separated into two subgroups--with or without multiple somatic symptoms---at the cut-off point of 10 [17]. Recruitment continued until equal number of patients was enrolled in two subgroups from each medical setting (*n* = 25).

### Subjects

The inclusion criteria of the study were as follows: age 18 years or above, seeking treatment voluntarily for their own problems, and being able to read and sign the informed consent form. The exclusion criteria included language barriers, limited writing skills, cognitive impairment/organic brain disorder/dementia, psychosis, and acute suicidal tendency. All patients were registered, including those who denied participation with reasons (such as lack of time, lack of interest in the study, lack of trust, etc.). Both research assistants (medical students) and clinical doctors ensured that the above criteria were fulfilled.

### Assessment instruments

Somatic symptom severity was measured with the PHQ-15. This instrument includes 15 prevalent somatic symptoms in primary care [18]. Studies in both Western and Chinese populations have demonstrated the satisfactory reliability and validity of the PHQ-15 [6, 17, 19, 20]. An optimal cut-off point of 10 was recommended to screen patients with somatoform disorders [17]. An additional question was asked about the symptom duration. The frequency of doctor visits in the past 12 months was also assessed.

The structured clinical interview ICAB was designed to assess the psycho-behavioral criteria associated with somatic symptoms. The development of this interview was introduced in Klaus’s cohort study [15], which has also demonstrated good relevance and predictive validity in the context of somatoform symptomatology. Eighteen items of with a binary response format (present/not present) were selected from the pool of 28 items that distinguished individuals with different levels of somatic symptom severity and health care utilization/impairment (see Additional file 1: Table S1) [14, 21]. Even though the interview and its nine-factor structure have been proved to be reliable and valid in patients with somatoform disorders, this should be the first time to investigating different operationalization of three dimensions of the SSD criteria B. No reference standard results were available to the participants or assessors when they completed the questionnaires.

The 7-item Whiteley Index (WI-7) [22] was used to evaluate illness anxiety. The Chinese WI-7 has proven to have satisfactory reliability and validity in a general Hong Kong population [23]. A cut-off score of 3 was recommended for screening hypochondriasis [24].

The 9-item depression scale of the Patient Health Questionnaire (PHQ-9) and the 7-item anxiety scale (GAD-7) were used to measure the severity of depression and generalized anxiety, respectively. Both of them have demonstrated good reliability and validity in screening for depressive and anxiety disorders in Chinese general hospital outpatients [25, 26].

The 12-item short form health survey (SF-12) captures reliable and valid information on health-related quality of life (QoL) in the Chinese population [27], resulting in a physical (PCS) and a mental composite score (MCS).

The Mini International Neuropsychiatric Interview Plus (MINI Plus, version 5.0.0) is a brief structured interview for the diagnosis of major axis I psychiatric disorders according to the DSM-IV diagnostic criteria [28]. The Chinese version of the MINI has been shown to have good reliability and validity [29]. In our study, modules listed in Table 1 were adopted to establish the diagnosis of somatoform disorders. All participants were invited to complete the MINI plus, which was carried out by trained research assistants.

**Table 1:** Modules utilized for the diagnosis of somatoform disorders according to DSM-IV

Somatoform disorders	
Pain disorder	
Hypochondriasis	
Somatization disorder	
Body dysmorphic disorder	

### Operationalization of the DSM-5 SSD concept

The assessment of SSD was operationalized as followed:

For criterion A, at least one physical symptom in the PHQ-15 had to be rated as “very bothering.”

For criterion C, symptoms had to last for more than 6 months to be rated as chronic.

For criteria B, the 18 items in the ICAB were classified as assessing the cognitive (B1), affective (B2), and behavioral (B3) subscales, as proposed by the theoretical conceptualization of DSM-5 SSD (see Additional file 1: Table S1). The cognitive subscale contains seven items to reflect disproportionate thoughts about the seriousness of somatic symptoms, such as “think about bodily complaints most of the time”, “hard to thank about other things”, “expect serious consequences”, etc. The affective subscale measures health anxiety with five items, such as “frequently worry about physical complaints, possible causes and consequences”, “worry a lot about health and possible illnesses” and so on. The behavioral subscale includes “frequent check bodily sensation”, “feeling of vulnerable or weak so as to avoid certain activities”, and “visit doctors as quickly as possible” to enrich the criteria of excessive time and energy devoted to somatic symptoms. The optimal cut-off points for each subscale were established to identify those with positive psycho-behavioral criteria. In addition, in order to compare the results with previous exploratory work [8, 30], the total WI-7 score was also employed with a cut-off point of 3 [24].

For the specification of the SSD severity, the mild type required that only one of the SSD B criteria can be fulfilled, the moderate type required two or more of the SSD B criteria, and the severe type required two or more of the SSD B criteria plus “multiple somatic complaints;” the latter were operationalized as a PHQ-15 ≥ 10 in our study.

### Statistical procedures

Categorical variables were described as absolute and relative frequencies and evaluated by chi-square difference tests. Continuous data were presented as the means and standard deviations, and they were compared by t-test for two independent groups and by one-way analysis of variance (ANOVA) for three or more independent groups. The Bonferroni method was adopted for multiple comparisons. Cohen’s кscores were given to illustrate the agreement of different diagnoses. Since 12 of the 491 (2.4%) participants had missing values, they were replaced with the mean value of the remaining items. A *p*-value of less than 0.05 (2-tailed) was considered significant.

Since equal numbers of patients with or without multiple somatic symptoms were recruited according to our study design, the proportion of SSD patients in the whole sample could not reflect their prevalence. As Schaefert et al.’s study found that 28.1% (79/281) of Chinese general hospital outpatients had a high somatic symptom severity (PHQ-15 ≥ 10) [6], the standardized rate of SSD in our study was calculated accordingly. For example, when 133/238 (55.9%) SOM+ patients and 51/253 (20.2%) SOM- patients in our sample fulfilled certain criteria, the prevalence would be estimated as 133/238*28.1% + 51/253*(1–28.1%) = 30.2%.

Cronbach’s α was used to estimate the internal consistency of the ICAB and its subscales. Confirmative factor analysis (CFA) was carried out to test its hypothesized factorial structure using the robust weighted least squares estimation with mean and variance adjustment (WLSMV) method. Fit indices based on the scaled chi-square statistic, such as the root mean square error of approximation (RMSEA) and comparative fit index (CFI), were used to evaluate the model fit. A value of 0.05 or less for RMSEA was considered to be very good, while 0.05–0.08 was acceptable and an RMSEA of up to 0.10 was mediocre (Browne and Cudeck, 1992). A value of 0.95 or greater for CFI was considered to be adequate (Bentler, 1990). Criterion validity was examined using the Spearman’s correlation between the ICAB total and subscale scores and total scores of the PHQ-15, PHQ-9, GAD-7, and WI-7. To operationalize the diagnostic criteria of SSD, the optimal cut-off points of the ICAB should be determined. Due to the lack of validated clinical interview of the SSD, we explored the potential cut-off points by both the percentages of screening-positive persons and the receiver operator characteristic (ROC) analysis with the quality of life serving as the reference standard.

Statistical analyses were performed with IBM SPSS Statistics 20.0 and Mplus version 7.0.

## Results

### Study sample and sociodemographic characteristics of participants

The detailed enrollment procedure has already been published [16]. A total of 799 patients were contacted, and 491 (61.4%) of them were included in the study. Two hundered thirty eight participants were classified as patients with multiple somatic symptoms (SOM+, PHQ-15 ≥ 10), with a mean age of 44.3 (±15.9) years and 73.6% being women. The comparison group (SOM-, PHQ-15 < 10) included significantly fewer women (57.9% vs. 73.6%, *p* < 0.001). There was no significant difference between SOM+ and SOM- participants in terms of other sociodemographic characteristics.

### Reliability and validity of the interview concerning cognitive, affective, and behavioral features (ICAB)

The ICAB has shown high reliability in this sample (Cronbach’s α = 0.90). The validity of the ICAB was assessed with the structure validity, criterion validity and known-group validity. Firstly, its three-factor structure was proved to be acceptable by the confirmative factor analysis (CFI = 0.962, RMSEA = 0.066, 90% confidence interval = 0.059–0.074). Secondly, the sum scores of the WI-7 served as an external validator, and showed moderate correlation with subscales of the ICAB (*r* = 0.42–0.61, *p* < .001). Finally, comparisons showed that patients with multiple somatic symptoms scored significantly higher than those without, both on the item level and subscale level of the ICAB (see Additional file 1: Table S1), indicating that the ICAB was valid to differentiate samples with positive psycho-behavioral characteristics.

Due to the lacking of golden standard, we explored the optimal cut-off points of the ICAB by the following two methods. First, we estimated the percentages of positive-screening participants at each cut-off point within each subscale. As shown in Table 2, if only one positive item was required, then the percentage of general hospital outpatients who fulfilled with the SSD criteria B would be as high as 91.4%. Those corresponding percentages decreased when the cut-off points for each subscale increased from 1 to 4.

**Table 2:** Operationalization of the SSD B criteria (*n* = 491)

	Total (*n* = 491)	SOM+ (PHQ-15 ≥ 10) (*n* = 238)	SOM- (PHQ-15 < 10) (*n* = 253)	*p*
SSD criterion B1 cognition (7 items)				
Total score	2.1 ± 1.9	2.7 ± 2.1	1.6 ± 1.5	*<.001*
Total score ≥ 1, n (%)	372 (75.8)	198 (83.2)	174 (68.8)	*<.001*
Total score ≥ 2, n (%)	288 (58.7)	161 (67.6)	127 (50.2)	*<.001*
Total score ≥ 3, n (%)	146 (29.7)	99 (41.6)	47 (18.6)	*<.001*
Total score ≥ 4, n (%)	105 (21.4)	76 (31.9)	29 (11.5)	*<.001*
SSD criterion B2 affects (5 items)				
Total score	2.1 ± 1.6	2.5 ± 1.7	1.7 ± 1.5	*<.001*
Total score ≥ 1, n (%)	368 (74.9)	198 (83.2)	170 (67.2)	*<.001*
Total score ≥ 2, n (%)	306 (62.3)	168 (70.6)	138 (54.5)	*<.001*
Total score ≥ 3, n (%)	173 (35.2)	110 (46.2)	63 (24.9)	*<.001*
Total score ≥ 4, n (%)	120 (24.4)	83 (3 4.9)	37 (14.6)	*<.001*
SSD criterion B3 behaviors (6 items)				
Total score	2.6 ± 1.7	3.1 ± 1.7	2.1 ± 1.6	*<.001*
Total score ≥ 1, n (%)	422 (85.9)	222 (93.3)	200 (79.1)	*<.001*
Total score ≥ 2, n (%)	337 (68.6)	186 (78.2)	151 (59.7)	*<.001*
Total score ≥ 3, n (%)	194 (39.5)	125 (52.5)	69 (27.3)	*<.001*
Total score ≥ 4, n (%)	137 (27.9)	90 (37.8)	47 (18.6)	*<.001*
SSD B criteria total score				
Total score	6.7 ± 4.6	8.2 ± 4.8	5.3 ± 3.9	*<.001*
At least one criterion ≥1, n (%)	449 (91.4)	230 (96.6)	219 (86.6)	*<.001*
At least one criterion ≥2, n (%)	391 (79.6)	208 (87.4)	183 (72.3)	*<.001*
At least one criterion ≥3, n (%)	246 (50.1)	149 (62.6)	97 (38.3)	*<.001*
At least one criterion ≥4, n (%)	191 (38.9)	123 (51.7)	68 (26.9)	*<.001*
WI-7 total score	3.7 ± 2.1	4.6 ± 2.0	2.9 ± 1.9	*<.001*
Total scores ≥3, n (%)	334 (68.0)	192 (80.7)	142 (56.1)	*<.001*

Note: *SOM+* patients with multiple somatic symptoms (PHQ-15 ≥ 10); *SOM-* patients without multiple somatic symptoms (PHQ-15 < 10). Italic values indicate significance of *p* value (*p* < 0.05)

**Fig. 1:**
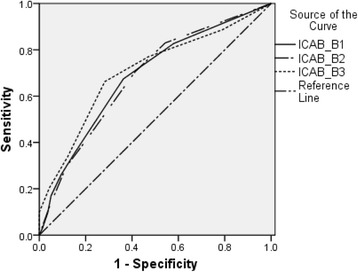
Diagnostic performance of the subscales of the ICAB for predicting the PCS

**Fig. 2:**
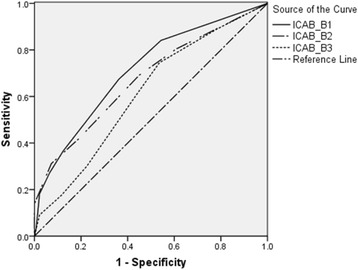
Diagnostic performance of the subscales of the ICAB for predicting the MCS

Then the ROC analyses with the quality of life serving as the reference standard were conducted (see Table 3, Figs. 1 and 2). The best diagnostic performances for PCS and MCS were both achieved at the cut-off points of 2 for the B1 and B2 subscales. For the B3 subscale, the optimal cut-off was 2 in predicting MCS and was 3 in predicting PCS.

**Table 3:** Cut-off points of the subscales of the ICAB (n = 491)

	SF-12/ PCS		SF-12/ MCS	
	Sensitivity	Specificity	Sensitivity	Specificity
Cut-off for SSD criterion B1 cognition (7 items)				
AUC (95% CI)	0.69 (0.63–0.74)		0.71 (0.66–0.76)	
Total score ≥ 1	0.83	0.42	0.84	0.46
Total score ≥ 2	*0.67*	*0.64*	*0.67*	*0.64*
Total score ≥ 3	0.35	0.85	0.37	0.88
Total score ≥ 4	0.26	0.91	0.27	0.94
Cut-off for SSD criterion B2 affects (5 items)				
AUC (95% CI)	0.68 (0.63–0.74)		0.68 (0.63–0.73)	
Total score ≥ 1	0.83	0.46	0.81	0.39
Total score ≥ 2	*0.71*	*0.59*	*0.69*	*0.55*
Total score ≥ 3	0.41	0.80	0.42	0.81
Total score ≥ 4	0.30	0.88	0.31	0.93
Cut-off for SSD criterion B3 behaviors (6 items)				
AUC (95% CI)	0.71 (0.66–0.76)		0.61 (0.55–0.67)	
Total score ≥ 1	0.89	0.21	0.89	0.22
Total score ≥ 2	0.77	0.53	*0.75*	*0.46*
Total score ≥ 3	*0.66*	*0.72*	0.60	0.57
Total score ≥ 4	0.34	0.88	0.30	0.78

Note: Italic values indicate results at the optimal cut-off points

Given all the above results, we recommend a cut-off point of 2 for three subscales, which means at least two items within either subscale should be positive to meet the SSD criteria B.

### Frequencies and agreement of different operationalization of the DSM-5 SSD criteria

For criterion A, 347/491 (70.7%) participants in our sample rated at least one physical symptom in the PHQ-15 as “very bothering”. Concerning criterion C, 338/491 (68.8%) participants reported that their complaints had lasted for more than 6 months. The standardized rates of SSD (fulfilling criteria A, B, and C) were estimated as 36.5% and 30.2% respectively, when the B criteria were operationalized with the ICAB and the WI-7 (Table 4). The agreement between the WI-7 and ICAB in diagnosing SSD was high (Cohen’s к = 0.769).

**Table 4:** Different operationalization and severity specifications of the DSM-5 SSD concept (n = 491)

	Total (*n* = 491)	Standardized rate (%)
Criteria A/ B (WI-7)/ C		
WI-7 ≥ 3, n (%)	184 (37.5)	30.2
Criteria A/ B (ICAB)/ C		
At least one B criteria ≥2, n (%)	215 (43.8)	36.5
Severity specification		
Mild type		
Only one of B criteria ≥2, n (%)	39 (7.9)	5.9
Moderate type		
Two or more B criteria ≥2 + SOM-, n (%)	59 (12.0)	16.7
Severe type		
Two or more B criteria ≥2 + SOM+, n (%)	117 (23.8)	13.8

### Agreement between DSM-IV somatoform disorders and DSM-5 SSD

**Fig. 3:**
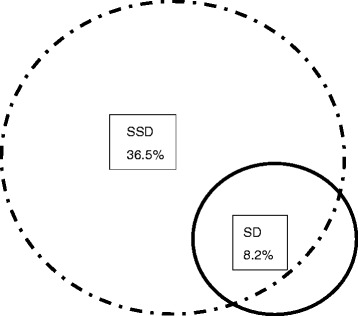
The agreement between DSM-IV somatoform disorders and DSM-5 SSD

According to the MINI interview, the standardized rate of somatoform disorders was estimated as 8.2%. The most common subtypes were hypochondriasis (3.3%), pain disorder (3.0%), somatization disorder (1.7%), and body dysmorphic disorder (0.7%). The Cohen’s Kappa between the diagnoses of DSM-IV somatoform disorders and DSM-5 SSD was only 0.152, indicating that the agreement between those two diagnostic concepts was small. However, as shown in Fig. 3, the standardized rate of somatoform disorders was only about one quarter of SSD, which might explain such small agreement between them. To be specific, 73.3% patients with somatoform disorders also met the SSD criteria, while only 19.0% SSD patients were diagnosed with somatoform disorders.

### Operationalization and validity of the DSM-5 SSD severity specification

According to the number of positive psycho-behavioral features and the severity of somatic symptoms, the standardized rates of the mild, moderate, and severe SSD subtypes were estimated as 5.9%, 16.7%, and 13.8%, respectively (see Table 4).

To test the validity of the SSD severity specifications, the sociodemographic and clinical features of non-SSD and SSD patients with different severity levels were compared. As shown in Table 5, it seemed that mild SSD patients had higher monthly family income, and higher percentages of mild and moderate SSD patients were employed, compared with the severe type and the non-SSD general hospital outpatients. There is no significant difference among them in terms of other sociodemographic features.

**Table 5:** Sociodemographic and clinical characteristics of the different SSD severity types (n = 491)

	Non SSD (*n* = 276)	Mild (*n* = 39)	Moderate (*n* = 59)	Severe (*n* = 117)	*p*
Age (M ± SD)	45.1 ± 16.9	42.9 ± 16.7	44.6 ± 16.3	45.3 ± 15.2	.876
Female (%)	63.1	69.2	61.0	71.8	.322
Insurance (yes %)	84.6	89.5	73.7	88.8	.057
Residence (%)					.217
City	82.5	76.9	71.2	77.8	
Rural	17.5	23.1	28.8	22.2	
Marital status (%)					.574
Single	20.1	12.8	22.4	20.7	
Married	61.9	59.0	56.9	64.7	
Divorced/ widowed	17.9	28.2	20.7	14.7	
Life situation (%)					.620
Alone	11.3	10.3	17.2	13.2	
With others	88.7	89.7	82.8	86.8	
Monthly family income (%)					*.029*
Less than 4000rmb	39.4	25.6	44.1	49.1	
4000-8000rmb	33.6	56.4	39.0	31.6	
More than 8000rmb	27.0	17.9	16.9	16.9	
Occupation (%)					*.004*
Employed/Student	34.8	56.4	51.7	37.4	
Unemployed	40.7	17.9	17.2	33.0	
Retired	24.4	25.6	31.0	29.6	
Education (%)					.195
Elementary	11.2	7.7	10.2	11.2	
Middle school	44.6	51.3	49.2	59.5	
University or higher	44.2	41.0	40.7	29.3	
PHQ-15 total score (M ± SD)	7.2 ± 5.2^1^	11.1 ± 3.5^2^	6.6 ± 2.1^1^	14.3 ± 3.4^3^	*<.001*
PHQ-9 total score (M ± SD)	7.5 ± 6.3^1^	8.7 ± 4.7^1^	8.7 ± 6.8^1^	13.0 ± 6.4^3^	*<.001*
GAD-7 total score (M ± SD)	5.2 ± 5.2^1^	4.0 ± 3.8^1^	6.5 ± 5.5^2^	9.5 ± 6.0^3^	*<.001*
WI-7 total score (M ± SD)	3.2 ± 2.0^1^	3.3 ± 1.8^1^	3.8 ± 1.8^2^	5.2 ± 1.8^3^	*<.001*
SF-12 PCS (M ± SD)	45.7 ± 7.7^2^	46.2 ± 7.9^2^	43.0 ± 6.7^1^	41.3 ± 6.4^1^	*<.001*
SF-12 MCS (M ± SD)	43.2 ± 11.0^2^	42.6 ± 10.7^2^	40.2 ± 9.6^2^	35.5 ± 12.1^1^	*<.001*
Doctor visits in the past 12 months (%)					*<.001*
0–2	41.0	35.9	22.8	16.5	
3–10	36.0	46.2	40.4	34.8	
11–20	8.8	2.6	17.5	20.9	
> 20	14.2	15.4	19.3	27.8	

Note: The Bonferroni method was adopted for multiple comparisons: values with^3^ were significant higher than values with^2^, and values with^2^ were significant higher than values with^1^ in multi-group comparison. Italic values indicate significance of *p* value (*p* < 0.05)

Non-SSD and SSD patients with different severity levels differed significantly regarding all clinical characteristics (see Table 5). Among them, the severe SSD patients consistently had the severest somatic, depressive, general anxiety, and health anxiety symptoms, the most impaired physical and mental QoL, and the most frequent doctor visits. Compared with the non-SSD group, patients with mild SSD had more somatic symptoms, but not more psychological, functional or behavioral problems. The level of anxiety, the QoL impairment, and the number of doctor visits of patients with moderate SSD was also “moderate,” that is, significantly higher than the corresponding measures in the mild type, but lower than those of the severe type. This supports that the operationalization of SSD severity was valid.

## Discussion

To the best of our knowledge, this is the first study to operationalize the DSM-5 SSD criteria with structured interview among general hospital outpatients.

Classified into the cognitive, affective, and behavioral subscales, the ICAB demonstrated high reliability and good structural validity in our clinical sample. Unlike our theoretically derived three-factor structure, a nine-factor structure was generated on the basis of exploratory factor analysis in Klaus’s study [15]. Since the three-factor structure was also valid and more compatible with the operationalization of SSD in our study, we adopted it in the subsequent analysis.

Since the diagnosis of SSD required only one of three psycho-behavioral criteria to be fulfilled, clinicians need to be particularly cautious with the interpretation of the descriptive adjectives of “disproportionate”, “high level” and “excessive”. As our results indicated, if only one positive item was required in the ICAB, as high as 91.4% of general hospital outpatients would be classified as met the SSD psycho-behavioral criteria. For clinicians, especially primary care practitioners, it might be difficult to judge to which degree should be regarded as pathological. Thus, a vast majority of patients could be under the risk of being mislabeled as with mental disorders, as some experts concerned [3]. Therefore, we strongly recommend using other measuring instruments, like the ICAB interview, to help to quantify the extent of abnormity. On one hand, based on our estimated percentages of positive-screening participants and the ROC analysis, at least two positive items within either subscale of the ICAB were required to fulfill the diagnostic criteria, which can help to mitigate the risk of mislabeling. On the other hand, our work enriched the diagnosis by including more cognitive, affective, and behavioral features confirmed by previously studies with somatization patients, so as to better represent the chronic and disabling somatoform symptomatology and to enhance diagnostic validity [16, 21].

Operationalized with the above standards of somatic symptoms, psycho-behavioral characteristics, and the duration, the proportion of SSD was estimated as 36.5% in Chinese general hospital outpatients. The prevalence of SSD was estimated as 51.8% among a sample of German psychosomatic inpatients [8], and as 47% in another sample of fibromyalgia syndrome patients [30]. And in a UK general population, 5.7% of participants reported both high somatic symptom burden and illness anxiety [31]. All three studies operationalized the psycho-behavioral criteria with the WI-14, which seemed to be a convenient and appropriate self-rated questionnaire, but could not measure disproportionate thoughts or behaviors related to somatic symptoms. Therefore, to obtain a clear picture of the prevalence of this new diagnostic concept, further studies with reliable diagnostic tools need to be conducted in different populations and countries. Nevertheless, those exploratory results warned us that the SSD diagnosis, if handled improperly, could become over-inclusive.

As far as we know, our study was also the first to examine the SSD severity specifications. Contrary to our expectation, the proportion of patients with mild SSD was the lowest. The reason for this might be that since the three features specified in the SSD B criteria were highly correlated, it was unlikely for a patient to have only one of them. Furthermore, the distribution might be different between outpatients and the general population, with subjects with mild symptom severity not consulting medical doctors. Further research in other settings is needed to clarify this.

Comparisons of sociodemographic features among SSD patients with different severity levels showed that mild SSD patients were better-off in terms of financial and employment status. The potential explanation for this phenomenon could be that their social function was less impaired. This reminded us the degree of social function impairment could be worthy of taken into account for the severity specifications of SSD. In addition, no gender difference was discovered between those with or without SSD. Nevertheless, past studies seemed to support a strong correlation between female gender and the high somatic symptoms severity [6, 16]. Such discrepancy could be caused by the different diagnostic criteria of DSM-5, or our operationalization methods. Further studies should be conducted in different populations and with different measurements to clarify the sociodemographic characteristics of patients with SSD.

Comparisons also revealed that the SSD severity subtypes were congruent with the level of depression, anxiety, quality of life impairment, and the frequency of doctor visits, which provided evidence for the validity of the operationalization of the SSD severity specifications. Similar to our results, a study from the Netherlands found that compared to patients with mild SSD, patients with moderate SSD suffered from lower physical functioning and higher levels of depression, while the levels of symptom severity and mental functioning were similar in both groups [9]. However, severe SSD was not defined in this study. Although no severity subtypes specified, a study by Voigt et al. also found that SSD was considerably associated with patients’ physical and mental function [32]. The possibility of identifying patients with different severity levels implies the importance of developing and evaluating a severity-stepped model of care.

Last but not least, similar as previous results [8, 9], our study found that the diagnostic agreement between the DSM-5 SSD and the DSM-IV somatoform disorders was small. In addition, we went a step further to clarify that the big difference between their estimated prevalence rates should be taken into consideration. Actually, most patients with somatoform disorders also met the SSD criteria, but only about one fifth SSD patients could be diagnosed with somatoform disorders. This was compatible with the enlargement of the diagnostic concept discussed above.

Our study has several limitations. 1) First, since the SCID-5 was still unavailable when our study was conducted, the diagnostic performance and the optimal cut-off point analyses of the ICAB had to be based on indirect indicators instead of the gold standard. 2) Second, equal numbers of patients with and without multiple somatic symptoms were recruited according to the study design instead of consecutively as a whole sample. Therefore, the percentages of SSD and the distribution of its subtypes could not reflect their real prevalence in Chinese general hospital outpatients. Nevertheless, the standardized rates were calculated based on the proportion of patients with multiple somatic symptoms in Chinese general hospital outpatients reported by our previous research. 3) Third, within the MINI Plus, there is no specific module for undifferentiated somatoform disorders; therefore, the prevalence of SD might have been underestimated, which could also influence the measurement of its agreement with SSD. 4) Finally, the results from our cross-sectional sample of Chinese general hospital outpatients from three typical medical settings (biomedicine, Traditional Chinese Medicine, and psychosomatic medicine) may not be generalizable to patients from other populations.

## Conclusions

We hope our work will enrich the understanding of the conceptualization of SSD and its operationalization for clinical practice and research. Our data showed that the operationalization of the diagnosis and severity specifications of SSD was valid, but the diagnostic agreement between DSM-5 SSD and DSM-IV somatoform disorders was small. Besides the shift of diagnostic focus, the much higher estimated prevalence rate of SSD should be taken into consideration. Our results suggested that the interpretation the SSD criteria should be made cautiously, so that the diagnosis would not became over-inclusive. Further research is necessary to examine the validity of the ICAB with the DSM-5 version of the SCID, explore the distribution of SSD in different populations, and finally develop and evaluate optimized management strategies for SSD of different severities.

## Additional file

Additional file 1:Table S1. Item loadings and the item and subscale level of the interview. The table presented the content of the ICAB interview, each item loadings, and the item and subscale level (of the SOM+ and SOM- subgroups and the total sample). (DOCX 26 kb)

## Abbreviations

GAD-7: the 7-Item anxiety scale
PHQ-15: the 15-Item somatic symptom scale of the patient health questionnaire
PHQ-9: the 9-Item depression scale of the patient health questionnaire
QoL: Quality of life
SD: Somatoform disorders
SF-12 MCS: Mental composite score of the 12-item short form health survey
SF-12 PCS: Physical composite score of the SF-12
SOM -: Patients without multiple somatic symptoms (PHQ-15 < 10)
SOM +: Patients with multiple somatic symptoms (PHQ-15 ≥ 10)
SSD: Somatic symptoms disorders
WI-7: The 7-item Whiteley Index

## References

[1] APA (1994). Diagnostic and statistical manual of mental disorders.

[2] APA (2013). Diagnostic and statistical manual of mental disorders.

[3] Frances A. The new somatic symptom disorder in DSM-5 risks mislabeling many people as mentally ill. BMJ. 2013;346:f1580. 10.1136/bmj.f1580 23511949

[4] Hsu LK, Folstein MF (1997). Somatoform disorders in Caucasian and Chinese Americans. J Nerv Ment Dis.

[5] Parker G, Cheah YC, Roy K (2001). Do the Chinese somatize depression? A cross-cultural study. Soc Psychiatry Psychiatr Epidemiol.

[6] Schaefert R, Honer C, Salm F, Wirsching M, Leonhart R, Yang J, Wei J, Lu W, Larisch A, Fritzsche K (2013). Psychological and behavioral variables associated with the somatic symptom severity of general hospital outpatients in China. Gen Hosp Psychiatry.

[7] Wong JY, Fong DY, Chan KK (2015). Anxiety and insomnia as modifiable risk factors for somatic symptoms in Chinese: a general population-based study. Quality of life research : an international journal of quality of life aspects of treatment, care and rehabilitation.

[8] Voigt K, Wollburg E, Weinmann N, Herzog A, Meyer B, Langs G, Lowe B (2012). Predictive validity and clinical utility of DSM-5 somatic symptom disorder--comparison with DSM-IV somatoform disorders and additional criteria for consideration. J Psychosom Res.

[9] van Dessel NC, van der Wouden JC, Dekker J, van der Horst HE (2016). Clinical value of DSM IV and DSM 5 criteria for diagnosing the most prevalent somatoform disorders in patients with medically unexplained physical symptoms (MUPS). J Psychosom Res.

[10] Barsky AJ (2016). Assessing the new DSM-5 diagnosis of somatic symptom disorder. Psychosom Med.

[11] Toussaint A, Murray AM, Voigt K, Herzog A, Gierk B, Kroenke K, Rief W, Henningsen P, Lowe B (2016). Development and validation of the somatic symptom disorder-B criteria scale (SSD-12). Psychosom Med.

[12] Herzog A, Voigt K, Meyer B, Wollburg E, Weinmann N, Langs G, Lowe B (2015). Psychological and interactional characteristics of patients with somatoform disorders: validation of the somatic symptoms experiences questionnaire (SSEQ) in a clinical psychosomatic population. J Psychosom Res.

[13] Klepac RK, Dowling J, Rokke P, Dodge L, Schafer L (1981). Interview vs. paper-and-pencil administration of the McGill pain questionnaire. Pain.

[14] Rief W, Mewes R, Martin A, Glaesmer H, Braehler E (2010). Are psychological features useful in classifying patients with somatic symptoms?. Psychosom Med.

[15] Klaus K, Rief W, Brahler E, Martin A, Glaesmer H, Mewes R (2015). Validating psychological classification criteria in the context of somatoform disorders: a one- and four-year follow-up. J Abnorm Psychol.

[16] Zhang Y, Fritzsche K, Leonhart R, Zhao X, Zhang L, Wei J, Yang J, Wirsching M, Nater-Mewes R, Larisch A (2014). Dysfunctional illness perception and illness behaviour associated with high somatic symptom severity and low quality of life in general hospital outpatients in China. J Psychosom Res.

[17] Korber S, Frieser D, Steinbrecher N, Hiller W (2011). Classification characteristics of the patient health Questionnaire-15 for screening somatoform disorders in a primary care setting. J Psychosom Res.

[18] Kroenke K, Spitzer RL, Williams JB (2002). The PHQ-15: validity of a new measure for evaluating the severity of somatic symptoms. Psychosom Med.

[19] Lee S, Ma YL, Tsang A (2011). Psychometric properties of the Chinese 15-item patient health questionnaire in the general population of Hong Kong. J Psychosom Res.

[20] Qian J, Ren Z, Yu D, He X, Li C (2014). Patient health questionnaire patient health Questionnaire-15(PHQ-15) reliability validity detection rate psychometrics. Chin Ment Health J.

[21] Rief W, Mewes R, Martin A, Glaesmer H, Brahler E (2011). Evaluating new proposals for the psychiatric classification of patients with multiple somatic symptoms. Psychosom Med.

[22] Fink P, Ewald H, Jensen J, Sorensen L, Engberg M, Holm M, Munk-Jorgensen P (1999). Screening for somatization and hypochondriasis in primary care and neurological in-patients: a seven-item scale for hypochondriasis and somatization. J Psychosom Res.

[23] Lee S, Ng KL, Ma YL, Tsang A, Kwok KP (2011). A general population study of the Chinese Whiteley-7 index in Hong Kong. J Psychosom Res.

[24] Conradt M, Cavanagh M, Franklin J, Rief W (2006). Dimensionality of the Whiteley index: assessment of hypochondriasis in an Australian sample of primary care patients. J Psychosom Res.

[25] He X, Li C, Qian J, Cui H, Wu W (2010). Reliability and validity of a generalized anxiety disorder scale in general hospital outpatients. Shanghai Archs Psychiatry.

[26] Xiong N, Fritzsche K, Wei J, Hong X, Leonhart R, Zhao X, Zhang L, Zhu L, Tian G, Nolte S (2015). Validation of patient health questionnaire (PHQ) for major depression in Chinese outpatients with multiple somatic symptoms: a multicenter cross-sectional study. J Affect Disord.

[27] Lam CL, Tse EY, Gandek B (2005). Is the standard SF-12 health survey valid and equivalent for a Chinese population?. Qual Life Res.

[28] Sheehan DV, Lecrubier Y, Sheehan KH, Amorim P, Janavs J, Weiller E, Hergueta T, Baker R, Dunbar GC (1998). The Mini-international neuropsychiatric interview (M.I.N.I.): the development and validation of a structured diagnostic psychiatric interview for DSM-IV and ICD-10. J clin psychiatry.

[29] Si T, Shu L, Dang W, Su Y, Chen J, Dong W, Kong Q, Zhang W (2009). Evaluation of the reliability and validity of chinese version of the Mini-international neuropsychiatric interview in patients with mental disorders. Chin Ment Health J.

[30] Hauser W, Bialas P, Welsch K, Wolfe F (2015). Construct validity and clinical utility of current research criteria of DSM-5 somatic symptom disorder diagnosis in patients with fibromyalgia syndrome. J Psychosom Res.

[31] Lee S, Creed FH, Ma YL, Leung CM (2015). Somatic symptom burden and health anxiety in the population and their correlates. J Psychosom Res.

[32] Voigt K, Wollburg E, Weinmann N, Herzog A, Meyer B, Langs G, Lowe B (2013). Predictive validity and clinical utility of DSM-5 somatic symptom disorder: prospective 1-year follow-up study. J Psychosom Res.

