# Supplementary material for: Operationalization of diagnostic criteria of DSM-5 somatic symptom disorders
Source: BMC Psychiatry. 2017 Nov 7;17:361. doi: 10.1186/s12888-017-1526-5 (PMC5678573; doi:10.1186/s12888-017-1526-5)
Supplement: Additional file 1:Table S1. — Item loadings and the item and subscale level of the interview. The table presented the content of the ICAB interview, each item loadings, and the item and subscale level (of the SOM+ and SOM- subgroups and the total sample). (DOCX 26 kb) [file 12888_2017_1526_MOESM1_ESM.docx]

Supplementary table 1. Item loadings and the item and subscale level of the interview

|  | Factor  loading | SOM+  Yes n (%)  (n=238) | SOM-  Yes n (%)  (n=253) | Total  Yes n (%)  (n=491) | *p* |
| --- | --- | --- | --- | --- | --- |
| **SSD criterion B1 cognitions** (7 items, Cronbach's α =.77) |  |  |  |  |  |
| **Total score** |  | 2.8±2.2 | 1.5±1.6 | 2.1±2.1 | **<.001** |
| 1. Do you think about bodily complaints most of the time during the day? **(Rumination)** | .81 | 85 (41.5) | 38 (18.3) | 123 (29.8) | **<.001** |
| 2. Is it hard for you to think about things other than bodily complaints? **(Lack of distraction)** | .63 | 51 (25.0) | 19 (9.1) | 70 (17.0) | **<.001** |
| 4. Do you expect serious consequences if your bodily complaints do not improve soon? **(Catastrophising of physical sensations)** | .87 | 113 (55.1) | 80 (38.5) | 193 (46.7) | **.001** |
| 7. If you start to think about pains and complaints, is it difficult to for you to stop these thoughts? **(Ruminations about physical complaints)** | .70 | 76 (37.3) | 37 (17.9) | 113 (27.5) | **<.001** |
| 8. If you experience bodily complaints or misperceptions, is your first thought that these are signs of serious illnesses (e.g., cancer, cardiac problems, diabetes)? **(Catastrophising of physical sensations)** | .78 | 93 (45.6) | 39 (18.8) | 132 (32.1) | **<.001** |
| 9. Are you firmly convinced that symptoms and pains are exclusively caused by physical conditions, even if doctors express other opinions? **(Somatic illness beliefs)** | .64 | 80 (39.2) | 44 (21.2) | 124 (30.1) | **<.001** |
| 17. Do you think it is unfair that you suffer from more illnesses than other people? **(Feelings of injustice)** | .64 | 70 (34.1) | 50 (24.0) | 120 (29.1) | **.024** |
| **SSD criterion B2 affects** (5 items, Cronbach's α =.81) |  |  |  |  |  |
| **Total score** |  | 2.6±1.8 | 1.6±1.6 | 2.1±1.8 | **<.001** |
| 5. Do you frequently worry about physical complaints and their possible causes and illness consequences? **(Worrying about physical complaints)** | .93 | 122 (59.5) | 80 (38.5) | 202 (48.9) | **<.001** |
| 6. Do you worry a lot about your health and possible illnesses? **(Illness worries)** | .85 | 127 (62.3) | 86 (41.3) | 213 (51.7) | **<.001** |
| 14. Do you frequently worry about your health? **(Health worries)** | .85 | 131 (64.2) | 83 (39.9) | 214 (51.9) | **<.001** |
| 15. Do your worry about suffering from a serious disease? **(Illness worries)** | .84 | 95 (46.6) | 53 (25.9) | 148 (36.2) | **<.001** |
| 16. Do you frequently despair because of physical complaints**? (Desperation because of symptoms)** | .76 | 57 (27.9) | 24 (11.5) | 81 (19.7) | **<.001** |
| **SSD criterion B3 behaviors** (6 items, Cronbach's α =.73) |  |  |  |  |  |
| **Total score** |  | 3.2±1.9 | 1.9±1.7 | 2.5±1.9 | **<.001** |
| 3. Do you frequently concentrate on bodily complaints to check whether they are changing? **(Bodily self-observation)** | .92 | 114 (55.6) | 71 (34.1) | 185 (44.8) | **<.001** |
| 10. Do you think that you are a very illness-vulnerable person who should try to avoid strain? **(Illness vulnerability)** | .68 | 110 (53.7) | 59 (28.4) | 169 (40.9) | **<.001** |
| 11. Do you think that you are so weak that you should avoid some or all everyday challenges**? (Negative self-concept of bodily weakness)** | .71 | 81 (39.5) | 40 (19.2) | 121 (29.3) | **<.001** |
| 12. Do you avoid any physical activities that could cause sweating or heart beat accelerations? **(Avoidance of physical activities that can provoke symptoms)** | .65 | 92 (44.9) | 61 (29.3) | 153 (37.0) | **.001** |
| 13. Do you try not to challenge some body parts because of bodily complaints? **(Disuse of body parts)** | .75 | 114 (55.6) | 60 (28.8) | 174 (42.1) | **<.001** |
| 18. In the case of physical complaints, do you prefer to visit doctors as quickly as possible to be sure about what kind of symptoms you have? **(inability to tolerate symptoms)** | .50 | 136 (66.7) | 112 (53.8) | 248 (60.2) | **.008** |

Note: p-value refers to non-parametric test for continuous variables with abnormal distribution and chi-square tests for categorical variables between the SOM+ and the SOM- sample, adjusted for the gender. SOM+: patients with multiple somatic symptoms (PHQ-15≥10); SOM-: patients without multiple somatic symptoms (PHQ-15<10); SSD: somatic symptoms disorders.
